# Structure of the Chloroplast Ribosome: Novel Domains for Translation Regulation 

**DOI:** 10.1371/journal.pbio.0050209

**Published:** 2007-08-07

**Authors:** Andrea L Manuell, Joel Quispe, Stephen P Mayfield

**Affiliations:** 1 Department of Cell Biology, The Scripps Research Institute, La Jolla, California, United States of America; 2 The Skaggs Institute for Chemical Biology, The Scripps Research Institute, La Jolla, California, United States of America; 3 National Resource for Automated Molecular Microscopy, The Scripps Research Institute, La Jolla, California, United States of America; Brandeis University, United States of America

## Abstract

Gene expression in chloroplasts is controlled primarily through the regulation of translation. This regulation allows coordinate expression between the plastid and nuclear genomes, and is responsive to environmental conditions. Despite common ancestry with bacterial translation, chloroplast translation is more complex and involves positive regulatory mRNA elements and a host of requisite protein translation factors that do not have counterparts in bacteria. Previous proteomic analyses of the chloroplast ribosome identified a significant number of chloroplast-unique ribosomal proteins that expand upon a basic bacterial 70S-like composition. In this study, cryo-electron microscopy and single-particle reconstruction were used to calculate the structure of the chloroplast ribosome to a resolution of 15.5 Å. Chloroplast-unique proteins are visualized as novel structural additions to a basic bacterial ribosome core. These structures are located at optimal positions on the chloroplast ribosome for interaction with mRNAs during translation initiation. Visualization of these chloroplast-unique structures on the ribosome, combined with mRNA cross-linking, allows us to propose a model for translation initiation in chloroplasts in which chloroplast-unique ribosomal proteins interact with plastid-specific translation factors and RNA elements to facilitate regulated translation of chloroplast mRNAs.

## Author Summary

Translation of mRNA into protein is the main step for the regulation of gene expression in the chloroplast, the photosynthetic organelle of plant cells. Translation is conducted by the ribosome, a large macromolecular machine composed of RNA and protein. Studies have shown that the composition of the chloroplast ribosome is similar to that of bacterial ribosomes, but also that chloroplast ribosomes contain a number of unique proteins. We present the three-dimensional structure of the chloroplast ribosome, as calculated using cryo-electron microscopy and single-particle reconstruction. Chloroplast-unique structures are clearly visible on our ribosome map, and expand upon a basic bacterial ribosome-like core. The role of these chloroplast-unique ribosomal proteins in regulating translation of chloroplast mRNAs, including light-regulated translation, is suggested by the location of these structures on the ribosome. Biochemical data confirm a predicted function in chloroplast translation for some of the unique proteins. Our model for translation in the chloroplast incorporates decades of biochemical and genetic studies with the structure presented here, and should help guide future studies to understand the molecular mechanisms of translation regulation in the chloroplast.

## Abstract 3

Cryo-electron microscopy and single-particle reconstruction were used to calculate the structure of the chloroplast ribosome. Chloroplast-unique proteins are visualized as novel structural additions to a basic bacterial ribosome core.

## Introduction

The chloroplast of plants and algae is believed to have originated from the endosymbiosis of an ancient photosynthetic bacteria into a eukaryotic host. Remnants of that ancient bacteria remain in the modern chloroplast, as it maintains a circular genome and transcription and translation machinery similar to that of prokaryotes [1,2]. Chloroplasts are responsible for photosynthetic energy production in plants and algae, and have recently been targeted as a platform for production of recombinant therapeutic proteins, making the understanding of translation in this organelle essential [3]. Approximately 60 proteins are translated in the plastid, a small fraction of the total proteins functioning in this organelle. The majority of chloroplast proteins are encoded by the nuclear genome and post-translationally imported into the plastid [4]. Coordinate expression from the nuclear and plastid genomes is required for development in photosynthetic organisms, and is achieved in chloroplasts primarily through regulation of translation [5,6]. Translation of many chloroplast genes is also regulated in response to light, and to maintain stoichiometric accumulation of multiprotein-complex subunits [7,8]. All of this regulation involves a host of protein translation factors, and the formation of RNA–protein complexes on chloroplast mRNA 5′ untranslated regions (5′ UTRs) [9–13]. Some of these protein factors are specific to individual mRNAs, whereas others serve classes of messages.

Due to the bacterial ancestry of the organelle, translation in the chloroplast has been considered bacterial-type translation, and many of the requisite bacterial-type translation factors can be identified in chloroplasts, although not all of these are exact homologs of the bacterial proteins [14]. Translation regulation in the chloroplast is more complex than in bacteria, and this complexity requires additional RNA and protein components not found in prokaryotic systems (reviewed in [5,15]). A number of protein factors have been identified as essential components of chloroplast translation, although how these factors interact with an mRNA to facilitate chloroplast translation is not known. Chloroplast messages also experience pausing during their translation, which has been implicated in maintaining the proper stoichiometry of gene expression from polycistronic mRNAs, as well as in cotranslational membrane insertion or cofactor association [16,17]. mRNA secondary structures or rare codon usage are often suggested as the cause of pausing during elongation; however, for mRNAs studied in chloroplasts (particularly *psbA* and *atpA*), these alone are insufficient to account for the pause sites.

RNA elements identified as regulatory components in the translation of chloroplast messages are primarily located in the 5′ UTR. These elements include Shine-Dalgarno (S-D) sequences, stem-loop structures, and A/U rich elements [10,18–20]. Nearly all bacterial mRNAs use base pairing between a S-D sequence located in the 5′ UTR of the mRNA and a complementary sequence located near the 3′ end of the 16S rRNA [21]. Base pairing between these sequences is essential for bacterial translation initiation, and bacterial S-D elements are located 7 ± 2 nucleotides (nt) upstream of the initiator AUG to allow for a simple physical positioning of the initiator AUG in the P-site of the ribosome [21,22]. In plastids, only some mRNAs have recognizable S-D sequences, and these are found over a large range of the 5′ UTR, some up to 100 nt upstream of the start site AUG [23,24]. This diverse positioning of S-D elements precludes a simple physical positioning of plastid mRNAs on the ribosome, and indicates that chloroplasts have a fundamentally different mechanism than bacteria for translation initiation.

**Figure 1:**
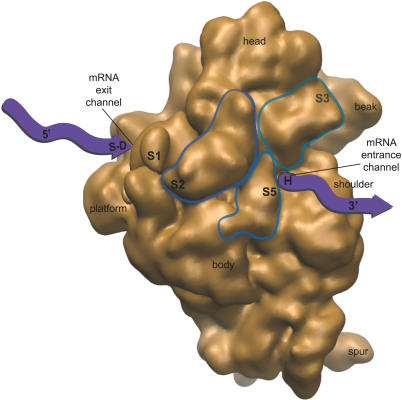
Predicted Location of Chloroplast-Unique Structures and Their Proximity to Functionally Important Regions of the Small Ribosomal Subunit The solvent-exposed surface of a bacterial small subunit [31] is shown and faces the reader. Locations of small subunit ribosomal proteins that have large additional domains on the chloroplast ribosome (S2, S3, and S5) [26] are circled. These proteins are labeled at the predicted location of chloroplast-unique domains, based on sequence homology with bacterial orthologs and bacterial ribosome structure; the S3 label is on the back side of the circled protein, facing the beak. mRNA (purple ribbon) enters and exits the small subunit through discrete channels that are the sites of important functions of the ribosome (S-D, S-D interaction; H, helicase activity; see text). Chloroplast-unique domains from S2, S3, and S5 are predicted to form a structural feature on the chloroplast ribosome that is positioned in proximity to mRNA-interacting regions of the small subunit.

The complete proteome of chloroplast ribosomes from both green algae [25,26] and higher plants [27,28] has been elucidated. A majority of the protein components of chloroplast ribosomes have clear homologs in bacterial 70S ribosomes. However, a significant number of chloroplast-unique proteins and domains were also identified (Tables S1 and S2). Five plastid-specific ribosomal proteins (PSRPs) have been identified in *Chlamydomonas reinhardtii,* four of which are located on the small subunit of the ribosome. Three other ribosomal proteins, S2, S3, and S5, have large chloroplast-unique domains on otherwise homologous bacterial ribosomal proteins [26]. Together, these protein additions increase the mass of the small subunit of the chloroplast ribosome by 25% compared to a bacterial 30S subunit (Table S1). Based on overall conservation of protein components and rRNAs, and the locations of proteins with chloroplast-unique domains (Figure 1), it has been hypothesized that novel structures have been added to the small subunit of the ribosome to accommodate the specific demands on chloroplast translation regulation [26].

In light of the accumulating evidence that translation regulation in the chloroplast is far more complex than in bacteria, and that chloroplast ribosomes contain unique protein components compared to 70S-type ribosomes, it is important to elucidate the structure of a chloroplast ribosome from the model organism most used to study chloroplast gene expression, C. reinhardtii. Using single-particle reconstruction from cryo-electron micrographs we have determined the structure of the C. reinhardtii chloroplast ribosome to a resolution of 15.5 Å. This structure shows that the chloroplast ribosome expands upon a core 70S-type bacterial ribosome structure with multiple chloroplast-unique domains. These chloroplast-unique structures are found on the small subunit of the ribosome near the mRNA entrance and exit channels. The potential role of these structures in translation regulation in the chloroplast is discussed, including their involvement in translation initiation via positioning of initiation mRNA–protein complexes (mRNPs), and the potential involvement of these unique domains in the processivity of chloroplast translation.

## Results

Chloroplast ribosomes from *C. reinhardtii,* a unicellular photosynthetic eukaryote, were isolated over successive sucrose gradients. Purified ribosomes were preserved in vitreous ice over continuous carbon grids and imaged using low-dose electron microscopy (see Figure S1A). Overall, chloroplast ribosome particles appear similar in structure to bacterial ribosome particles, as was anticipated from the homology of chloroplast and bacterial rRNAs and proteins. However, even in two dimensions, some orientations show evidence of structural addition to the chloroplast ribosome not found on the bacterial ribosome (Figure S1C). Starting from a dataset of over 100,000 particles, reference-free classification and hierarchical clustering allowed for the selection of the most homogeneous dataset totaling 42,934 particles (Figure S1B).

### Overall Structure and Comparison to 70S Ribosomes

**Figure 2:**
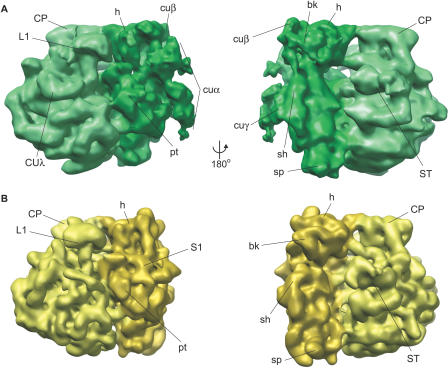
Three-Dimensional Map of the C. reinhardtii Chloroplast Ribosome at 15.5 Å (A) Chloroplast ribosome (green). Compared to (B) bacterial ribosome cryoEM structure (yellow; [29]). Left and right views are related by 180° rotation as indicated. At left is a classic side view of the ribosome with the large subunit on the left and the small subunit on the right; right view shows the small subunit on the left and the large subunit on the right. Common ribosome landmarks as well as chloroplast-unique structures are clearly defined on the chloroplast ribosome, and have been labeled. bk, beak; CP, central protuberance; cuα, cuβ, cuγ, and CUλ, chloroplast-unique structures; h, head; L1, L1 arm; pt, platform; sh, shoulder; sp, spur; ST, stalk. See Video S1 for a three-dimensional view of the chloroplast ribosome structure.

Single-particle reconstruction was used to calculate a three-dimensional map of the chloroplast ribosome to a resolution of 15.5 Å (as determined by 0.5 cutoff Fourier Shell Correlation criteria; Figure S2). The chloroplast ribosome has clearly defined large and small subunits, and a distinct intersubunit space (Figure 2 and Video S1). Common features defined from bacterial 70S ribosome structures can also be identified on the chloroplast ribosome; the large subunit of the chloroplast ribosome has an easily distinguished central protuberance (CP in Figure 2A), L1 arm (L1), and stalk (ST). The small subunit has clear head (h), body (b), platform (pt), shoulder (sh), and spur (sp) domains. The small subunit also has clearly distinguishable additional structures on its solvent-exposed face that are not present on bacterial ribosomes; these include a large multilobed structure emerging in the vicinity of the mRNA exit channel and extending down across the body of the ribosome (cuα, Figure 2A), additional connection of the head and beak regions (cuβ), and a thickening of the shoulder region (cuγ). The platform is also lifted slightly away from the body and towards cuα. On the large subunit, chloroplast-unique density extends from the base of the L1 arm back towards the center of the large subunit (CUλ). There is also extensive connection between the L1 arm and nearby features on the body of the large subunit, but this is also seen to some degree in the Escherichia coli cryo-electron microscopy (cryoEM) maps (Figure 2B; [29,30]) and represents flexibility inherent to this part of the large subunit.

Overall, interface surfaces and the tRNA and essential translation factor-binding regions located between the large and small subunits of the chloroplast ribosome appear highly similar with these same features in bacterial ribosomes, whereas solvent-exposed surfaces of the chloroplast ribosome show some striking differences from those of bacteria. It is difficult to distinguish individual bridges (as defined in [29] and [31]) in the central region of the intersubunit space (i.e., bridges B3 and B5), but the bridging patterns appear conserved between E. coli and the chloroplast ribosome, with only a few exceptions. Bridges 1b and 1c are seen as a single bridge off the upper central protuberance, and bridge 4 is expanded in the chloroplast ribosome and makes contact with the lower body region of the small subunit (unpublished data). The identification of these conserved bridges supports the idea that interactions between the large and small subunits are largely unchanged between chloroplast and bacterial ribosomes.

**Figure 3:**
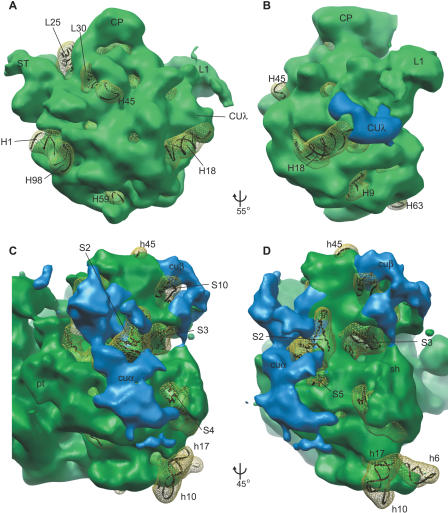
Structural Differences between Chloroplast and Bacterial Ribosomes Shown are differences between chloroplast and E. coli large ([A] and [B]) and small ([C] and [D]) subunits highlighting chloroplast-unique structures in blue, and E. coli ribosome densities lacking from the chloroplast ribosome in yellow mesh. Ribbon underlay of X-ray structure allows identification of missing densities. Landmarks as in Figure 2, plus some helix (e.g., H45) and protein (e.g., L25) labels. See text, Figures S3 and S4, and Tables S1 and S2 for specifics on these helices and proteins. (A) Solvent-exposed face of the large subunit. Helices and proteins that are not present on the chloroplast ribosome are clearly identified in difference density. (B) Rotated from (A) as indicated. The largest chloroplast-unique density on the large subunit is at the base of the L1 arm (CUλ). (C and D) Solvent-exposed face of the small subunit. See text for a discussion of each main chloroplast-unique structure (cuα and cuβ).

Normal mode flexible fitting was used to fit bacterial ribosome crystal structure data to our chloroplast ribosome map (see Materials and Methods). A difference map between this fitting and our chloroplast map reveals densities both unique to (cu structures) and lacking from (mesh/ribbon in Figure 3) the chloroplast ribosome. A majority of the densities lacking from the chloroplast ribosome can be understood in light of proteomic and genomic data on the chloroplast ribosomal proteins [25] and comparison of predicted rRNA secondary structures (Comparative RNA Web Site, http://www.rna.ccbb.utexas.edu; Figures S3 and S4). For example, large subunit proteins L25 and L30 are clearly identified in a difference map as lacking from the chloroplast ribosome (Figure 3A). These proteins were not identified in proteomic analysis of the C. reinhardtii chloroplast ribosome, nor were genes encoding these proteins identified in the completed C. reinhardtii nuclear genome sequence (http://genome.jgi-psf.org/Chlre3/Chlre3.home.html). There is no known function for either of these proteins on the ribosome [32]. The L29 protein was not identified via proteomics [25], and an L29 homolog has yet to be found in the C. reinhardtii genome database, but density in the area where this protein is found on the bacterial ribosome is clearly present in the chloroplast ribosome structure. L29 is involved in interactions with trigger factor and SRP, both of which have homologs in the C. reinhardtii chloroplast [33,34]. It is likely that the small size of L29 precluded its identification via proteomics, and that remaining gaps in the genome sequence are harboring the gene for chloroplast L29.

Small rRNA helices are also lacking from the chloroplast ribosome in a number of places on both the small and large subunits (Figure 3). In each case, the absence of rRNA density corresponds to a small region of the rRNA that is not conserved between chloroplasts and bacteria (Figures S3 and S4). The regions of rRNA that differ between chloroplast and bacteria are not involved in any known function of the ribosome; they do not interact with antibiotics, are not associated with any aspect of translation initiation, and do not participate in intersubunit bridges [35–38]. Comparison with predicted secondary structure diagrams from both mitochondrial and 80S ribosomes indicates that all of these helices are found in regions of variability off the conserved rRNA core shared by all ribosomes [39]. The identification of structural differences that correspond exactly with our previous proteomic analysis and with predicted rRNA secondary structure differences gives us a very high degree of confidence that the map of the chloroplast ribosome that we have calculated is correct, and validates the chloroplast-unique densities that we identify as real and significant.

Comparison of the chloroplast ribosome with cryoEM reconstructions of the E. coli ribosome reveals that the head of the chloroplast ribosome is rotated and tilted away from the large subunit by approximately 5°, which results in a slight lift of the beak (Figure S5). Connectivity between the beak and the shoulder in this area originates from chloroplast-unique density (cuβ), whereas connections are only seen between the beak helix and the shoulder in E. coli ribosomes. This is similar to movements seen in eukaryotic ribosomes upon internal ribosome entry site (IRES) binding (see Discussion). Similarity to the mitochondrial ribosome is also observed in the differences between chloroplast and bacterial large subunits. Like the chloroplast ribosome, mitoribosomes do not have L25, and there is a crevasse between the central protuberance and the stalk side of the large subunit where this protein sits in bacteria [40]. This effect is smaller but similar on the chloroplast ribosome since the central protuberance is much expanded on the mitoribosome.

In bacteria, the ribosomal E-site is commonly occupied by tRNA after purification, but we do not see this for our chloroplast ribosome. There is some evidence of partial occupancy at the factor-binding site of the chloroplast ribosome. The discontinuous density in the factor-binding site is not shown here, but may contribute to regions of large subunit density in the stalk base area and small subunit density on the back of the shoulder that appear extended into the intersubunit space, and also to cuɛ on the PSRP-7 antibody-bound map (see below). Further computational separation of a larger dataset may allow us to calculate a map representing full occupancy at this site, and further proteomic analysis could verify the identity of the bound factor.

### Structure near the mRNA Exit Channel Binds mRNA

**Figure 4:**
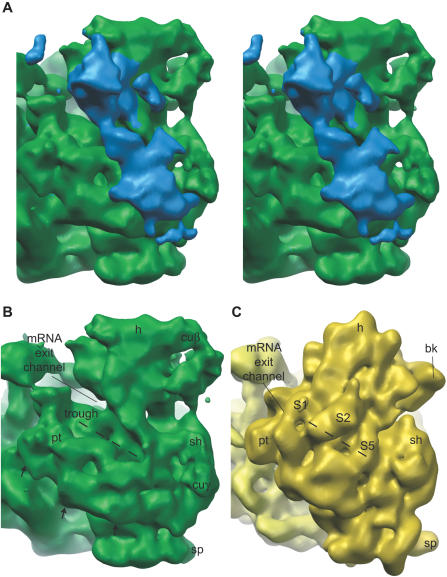
Chloroplast-Unique Structures Dominate the mRNA Exit Channel Area Solvent-exposed face of the small subunit is shown slightly turned to look at the mRNA exit channel. (A) Stereo pair image. The largest chloroplast-unique structure on the ribosome, cuα (blue), emerges from the head and neck of the small subunit and partially defines the path of access for mRNA at the exit channel. (B) cuα has been removed from this image to reveal the trough from the mRNA exit channel down into the midbody of the small subunit (dashed line). The platform is slightly lifted on the chloroplast ribosome, as indicated by the black arrows (arrows not to scale), accentuating the trough. (C) E. coli ribosome cryoEM structure for comparison indicates the location of proteins known to be altered in the chloroplast ribosome along the path of the trough (S1, S2, and S5; see Figure S3).

Chloroplast-unique structures and changes near the mRNA exit and entrance channels dominate a comparison of E. coli and chloroplast ribosomes (Figures 3 and 4). Connectivity with rRNA or proteins that have bacterial homologs allows prediction of the identity of some of the novel structures found on the chloroplast ribosome. The largest region of chloroplast-unique density on the small subunit emerges from the neck region of the ribosome, adjacent to the mRNA exit channel, and extends down along the platform (cuα; Figure 4A). This multilobed structure of approximately 90 kDa makes contact with the head and neck of the small subunit, and partially overlaps the positions of S1 and S2 on bacterial ribosomes (compare Figure 4A and 4C). The upper lobe of cuα limits access to the mRNA exit channel to about 25 Å from both side and top. The mRNA exit channel is the site of initial interactions between mRNAs and the ribosome; and in bacteria, this is the site of the S-D interaction that positions the start site AUG of the mRNA at the P-site of the ribosome [21,22,41,42]. Below cuα at the mRNA exit channel and following down the underside of the chloroplast-unique density, there is an extended trough on the chloroplast ribosome, accentuated by the lifting of the platform domain (Figure 4B). Proteins S21, near the mRNA exit channel, and S1, S2, and S5 are partially displaced from their positions on the bacterial ribosome by the chloroplast ribosome trough. These displacements may indicate movement of these proteins into cuα.

**Figure 5:**
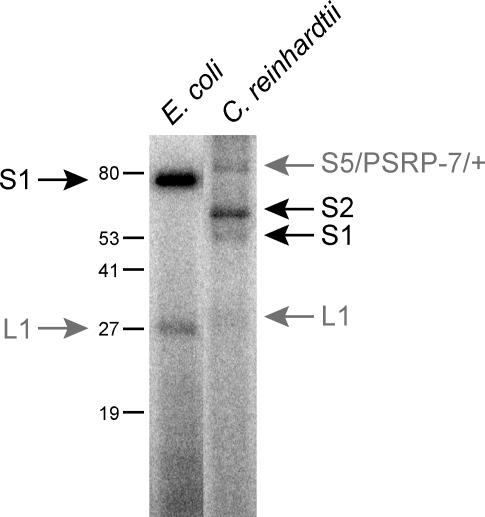
Cross-Linking of Plastid mRNA 5′ UTR to Chloroplast and E. coli Ribosomes Radiolabeled *psbA* 5′ UTR was incubated with purified chloroplast or E. coli ribosomes, UV irradiated to cross-link mRNA, and then proteins were separated by SDS-PAGE. Protein size markers are indicated (in kDa). In *E. coli,* S1 is the main protein that is cross-linked to mRNA, while a small amount is cross-linked to another protein, presumably L1 (gray arrow on left lane). Chloroplast ribosomes have two proteins that clearly bind to mRNA, S1 and S2; two other bands are also labeled (gray arrows on right lane). One of these bands is L1, and the other appears as an incompletely denatured protein complex containing at least S5 and PSRP-7. Mass spectrometry was used to identify the protein component of labeled chloroplast ribosome bands (see Materials and Methods).

Connectivity of cuα with the main body of the small subunit of the ribosome suggests that S1 and the chloroplast-unique domain of S2 comprise the majority of cuα. Chloroplast S1 is the only small subunit protein that is significantly smaller than its bacterial homolog (Table S1), but like bacterial S1, binds mRNA [43]. Chloroplast S2 has a large chloroplast-unique amino-terminal extension [26], more than doubling its size compared to bacterial S2 (63 kDa vs. 27 kDa, Table S1); two TRAM domains in this addition give chloroplast S2 the potential to bind RNA [44]. In ultraviolet (UV) cross-linking experiments, both of these proteins are strongly labeled by a radiolabeled mRNA 5′ UTR (Figure 5). Also labeled were L1 and an incompletely denatured protein complex containing at least S5 and PSRP-7, the other two large chloroplast-unique proteins on the small ribosomal subunit. In the same experiment using E. coli ribosomes, only S1 is strongly labeled (Figure 5).

The mass of cuα is estimated at 90 kDa, which adds over 10% greater mass to the small subunit of the chloroplast ribosome compared with the E. coli 30S subunit, and allows for S1 (44 kDa) and S2 to be contained within this structure. Given proximity to the mRNA exit channel and the RNA-binding properties of S1 and S2, cuα is perfectly situated to act as a landing pad for chloroplast initiation complex mRNPs. Interaction between these mRNPs and the ribosome could be utilized to position mRNAs, both with and without S-D sequences, for translation initiation.

### Chloroplast-Unique Density Approaches the mRNA Entrance Channel

**Figure 6:**
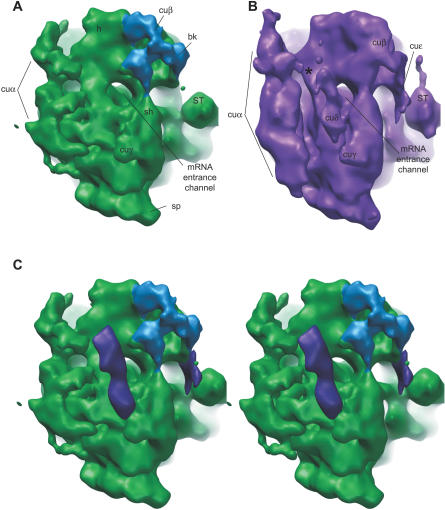
Chloroplast-Unique Density Approaches the mRNA Entrance Channel Solvent-exposed face of the small subunit is shown slightly turned to look at the mRNA entrance channel. (A) cuβ (blue) connects the beak helix with the head, squaring out this normally pointed feature on the ribosome, and approaches the top of the mRNA entrance channel. Connection to the shoulder stems from this chloroplast-unique structure as well. (B) PSRP-7 antibody-labeled chloroplast ribosome structure has additional density near the mRNA entrance channel (cuδ) and extending from the beak helix toward the factor-binding site (cuɛ). (C) Stereo pair image (putative PSRP-7 density labeled in purple) shows the position of cuδ across the mRNA entrance channel and paralleling cuα off the solvent-exposed face of the small subunit.

Another large region of chloroplast-unique density on the small subunit is found in the beak and head region of the ribosome, adjacent to the mRNA entrance channel (cuβ; Figure 6A). This is the first surface of the ribosome that coding regions of mRNA encounter during translation, and proteins in this region are important for helicase activity of the bacterial ribosome [45]. cuβ connects the beak helix (h33) with S3 and S10 (see Figure 3C), and approaches the mRNA entrance channel at the front underside of the beak. Chloroplast S3 has a large internal chloroplast-unique domain, as well as good homology with bacterial S3 at its N- and C-termini, which together predict that the S3 chloroplast-unique domain comprises cuβ (compare predicted location of S3 cu domain in Figure 1 with cuβ in Figure 6).

In an attempt to localize the largest plastid-specific ribosomal protein, PSRP-7, chloroplast ribosomes were incubated with PSRP-7 antibody prior to freezing and imaging. A separate reconstruction from this data yielded a map at 19.4 Å, and revealed additional structure on the solvent-exposed face of the small subunit (Figure 6B and 6C). Most of this additional density stems from chloroplast-unique structures already defined on the unliganded chloroplast ribosome, which are expanded in this antibody-bound map. A few regions of density that do not correspond to densities on the unliganded structure may represent the bound antibody (Figure 6C). These densities are found both emerging from the expanded shoulder of the small subunit (cuδ), and from the head of the small subunit and towards the factor-binding site at the subunit interface (cuɛ). cuɛ may also be related to the protein occupancy at the factor-binding site, because visualization at lowered thresholds reveals that cuɛ is contiguous with density in the factor-binding site.

Antibody-binding appears to stabilize chloroplast-unique structures on the small subunit, particularly cuα, and at very low thresholds connects the mid-region of cuα with the tip of cuδ (asterisk in Figure 6B). The tip region of cuδ is visualized at very low thresholds on the unliganded chloroplast ribosome map, which further suggests that antibody binding is stabilizing part of the chloroplast-unique density on the surface of the small subunit. cuδ extends toward the head parallel to cuα, and lies across the line of direct access to the mRNA entrance channel (Figure 6C). Chloroplast-unique structures near the mRNA entrance channel—via S3, which is involved in helicase activity in bacterial ribosomes [45], or PSRP-7, which binds mRNA (Figure 5 and [46])—are likely involved in recognizing structured elements in coding regions of chloroplast mRNAs and may act to alter the processivity of translation. These structures may also be involved in mRNA positioning for translation initiation, in analogy to the mRNA gate structure on the mitochondrial ribosome [40]. Mammalian mitochondrial mRNAs do not have 5′ UTRs [47,48], and the mRNA gate is hypothesized to function in the proper positioning of these leaderless messages for translation initiation [40].

Antibody-binding stabilization of at least cuα indicates that there is flexibility in these chloroplast-unique structures and that, because of this flexibility, the full extent of these features is not yet resolved in our structure. Lowering the threshold visualization levels of either the bound or the unbound maps reveals additional density near the mRNA exit channel, extending up along the head and occluding access to the channel (unpublished data), suggesting that alterations in this area must occur to provide mRNAs access to the small subunit of the ribosome. Chloroplast ribosomes imaged in complex with mRNA, tRNA, and protein factors may be needed to fully resolve structures in this area.

## Discussion

This is the first report of the structure of a chloroplast ribosome, and comes from the organism from which the majority of information on plastid translation has been derived *(C. reinhardtii)*. The translation machinery in chloroplasts is clearly based on a prokaryotic-like core, though translation in eukaryotic plastids is more complex than in bacteria from both regulatory and physical perspectives. A large body of research has shown that translation is the key regulated step in chloroplast gene expression [49]. These studies have identified many key events in chloroplast gene expression: interactions of individual photosynthetic proteins with their own and partner protein mRNAs, the formation of mRNPs between nuclear-encoded translation factors and the 5′ UTRs of mRNAs, and the effects of light-induced signals on mRNP formation and translation initiation [7,9,10,12,13,50–52]. Structural analysis of the chloroplast ribosome and identification of chloroplast-unique structures on the ribosome provide an important understanding of the physical components utilized for translation regulation in this organelle. Chloroplast-unique structures dominate the solvent-exposed face of the small subunit, and approach both the mRNA entrance and exit channels. These structures are ideally situated to regulate translation initiation, and genetic and biochemical data suggest that these structures accompany and complement the use of modified S-D sequences and translation initiation mRNP formation.

Proteomic studies identified chloroplast-unique ribosomal proteins, primarily on the small subunit of the ribosome [26] (Tables S1 and S2). The structure presented here allows us to visualize these chloroplast-unique proteins as novel structural domains on the chloroplast ribosome. The large subunit of the chloroplast ribosome differs from the bacterial 50S subunit by only a few proteins, and we see only one significant chloroplast-unique region on this subunit (CUλ; Figures 2A and 3B). The primary function of the large subunit of the ribosome is peptide bond formation, and this most basic function of the ribosome has been conserved between eukaryotic, bacterial, and organellar ribosomes. Here, we confirm this expected structural conservation in the core of chloroplast ribosomes.

The small ribosomal subunit is responsible for interactions with mRNAs and initiation factors that position messages for translation initiation [21], and it also has the duty of quality control in codon decoding during translation. Regions of the small subunit that are responsible for decoding and quality control are structurally conserved with bacterial ribosomes, whereas the chloroplast-unique additions are seen on the small subunit of the ribosome in areas that intersect the path of mRNA during translation initiation, the key regulated step of chloroplast translation. The large chloroplast-unique structure found near the mRNA exit channel and extending down along the platform of the small subunit (cuα; Figures 2–4) is located near the site of binding for S1 in bacteria. S1 is the only ribosomal protein to bind mRNAs in bacteria, and it binds to mRNAs and the ribosome through six repeats of an RNA-binding motif; the S1 protein in chloroplasts has only three RNA-binding motifs. The additional domains on S2 and the chloroplast-unique protein PSRP-7 both contain RNA-binding domains that may complement the smaller chloroplast S1 protein in mRNA binding (Figure 5). The S-D interaction between bacterial mRNAs and the 16S rRNA also occurs in the mRNA exit channel area, and functions to position mRNAs for translation initiation. As mentioned previously, S-D sequences in chloroplast mRNAs do not share the bacterial consensus spacing from the start site AUG. This difference in spacing requires a fundamentally different mechanism for bacteria and chloroplasts to position mRNAs for translation initiation, and suggests that the additional chloroplast-unique structure located at the mRNA exit channel may function as adapters that positions chloroplast mRNAs properly for initiation. cuα is perfectly situated to function as this adapter for interactions between chloroplast initiation complex mRNPs and the chloroplast ribosome.

Programmed pausing has been observed in the translation of several chloroplast mRNAs, and in the case of D1 protein, this pausing is intimately associated with assembly of the nascent polypeptide with cofactors and partner subunits into the thylakoid membrane [16]. In bacteria, side chains from S3 form part of the lining of the mRNA entrance channel [22], and mutation to S3 affects the ability of the bacterial ribosome to unwind downstream coding-region secondary structure for ribosome translocation along an mRNA [45]. Interactions between coding regions and 5′ UTRs of chloroplast mRNAs also impact translation efficiency, and these interactions can be modified by proteins binding to the 5′ UTR of the mRNA [53,54]. The locations of cuβ and cuδ near the mRNA entrance channel (see Figure 6), combined with the mRNA-binding properties of PSRP-7, allow for interactions between these ribosomal proteins and coding regions of mRNAs that may assist positioning during translation initiation, or that recognize structured elements in coding regions of mRNAs and modify processivity during translation elongation. That cuɛ reaches from the beak into the factor-binding site, and that elongation factor Ts is covalently linked to the ribosome through PSRP-7 in many chloroplasts (as the PETs polyprotein [46]), suggest possible involvement in programmed pausing through modification of ribosome function in this important region.

Modified ribosome structures that are thought to impact translation initiation have also been identified in other organisms. A large structural element was found adjacent to the platform on the small subunit of the 80S-type ribosome from trypanosome [55]. The rRNA responsible for this structure is found in expansion segments of the small subunit rRNA that are found only in trypanosomes. Trypanosome mRNAs are also unique in that they all receive the same 5′ UTR through transsplicing, and interactions between the 5′ UTR and the ribosome are required for translation [56]. The novel structure on the trypanosome ribosome is implicated in translation initiation by virtue of its proximity to the mRNA exit channel, and also its potential to interact through base pairing with conserved regions in the trypanosome mRNA 5′ UTR [55]. The structure of the hepatitis C virus (HCV) IRES element complexed to the human 40S ribosomal subunit also revealed a structure similarly situated to cuα [57]. This IRES is used for positioning viral mRNAs with their start site AUG at the P-site of the ribosome in the absence of cellular translation factors [58]. The collective movements of the 40S subunit head upon IRES binding are quite similar to those seen between chloroplast and bacterial ribosomes. It has been suggested that these movements promote formation of preinitiation complexes in the absence of canonical initiation factors [57,59]. Each of these systems has highly regulated translation initiation, which suggests that structural adaptation to the ribosome for specialized translation regulation may be quite ubiquitous in nature. The small subunit of the ribosome has evolved and adapted as a means to regulate translation initiation, whereas the large subunit of the ribosome is more evolutionarily stable, maintaining the basic function and integrity of the core reactions of peptide bond formation and nascent peptide delivery.

**Figure 7:**
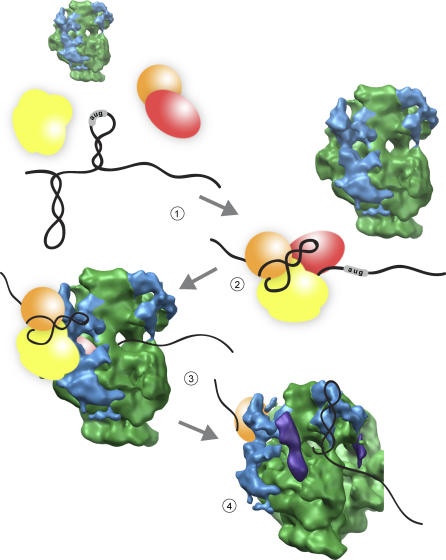
Translation Initiation in the Chloroplast Chloroplast mRNAs are not competent for translation initiation until assembled with mRNA-specific translation factors (panel 1). Translation factors assembled on the 5′ UTR of a chloroplast message modify the secondary structure of the mRNA and form a novel translation initiation mRNP (panel 2). This mRNP complex binds to the chloroplast ribosome through interaction with the chloroplast-unique structure (cuα, shown in blue) around the mRNA exit channel (panel 3). This binding positions the start site AUG of the mRNA in the P-site for translation initiation. During elongation, signal elements in the mRNA may be recognized by chloroplast-unique structures around the mRNA entrance channel like cuβ (panel 4). Such binding may modify the processivity of translation of that mRNA, and facilitate pausing to allow for cotranslational membrane insertion or cofactor assembly.

The chloroplast ribosome structure and mRNA cross-linking presented here have allowed us to propose a mechanistic model for chloroplast translation initiation (Figure 7). Such a model for bacterial translation is quite simple (as in Figure 1): mRNAs, even as they are being transcribed, are positioned via S1 binding and the S-D interaction with their start site AUG in the P-site of the ribosome ready for initiation. For this reason, bacterial initiation is dominated by accessibility of the S-D element and its physical spacing from the start site AUG. Plastid mRNAs are normally unable to access the ribosome on their own (Figure 7, panel 1), and require binding of nuclear-encoded proteins to activate translation (panel 2). We propose that cuα acts as a landing pad for initiation complex mRNPs as a first stage of mRNA interaction with the chloroplast ribosome (panel 3). Via this interaction, mRNAs with divergently spaced S-D sequences, or without S-D sequences, can be placed such that their start site AUG is correctly positioned in the ribosomal P-site for translation initiation. Interactions between chloroplast mRNA coding regions and 5′ UTRs may be sensed or accommodated by cuβ and cuδ near the mRNA entrance channel, and these domains may also assist in positioning of the start site AUG for initiation. Additionally, during translation, these chloroplast-unique structures may recognize sequence-specific or secondary-structured mRNA elements (Figure 7, panel 4) and communicate this to the ribosome in the form of modification of the processivity of translation or translocation. Such interactions would explain programmed pausing during translation that allow for proper membrane or cofactor association of nascent polypeptide chains.

The chloroplast ribosome structure presented here will allow for focused experimental design to examine interactions of ribosomal proteins and plastid mRNAs, as well as the role that these interactions play in translation initiation in the chloroplast. A clearer picture of the physical interactions involved in translation initiation—between mRNAs, their associated proteins, and the chloroplast ribosome—will also assist in designing more appropriate transgenes for increased recombinant protein expression in chloroplasts. This structure will also serve as a complement to studies on the basic mechanisms of translation that have traditionally used bacteria as a model.

## Materials and Methods

### Chloroplast ribosome purification.

C. reinhardtii cultures, strain cc3395, were grown to mid-log phase, harvested, and disrupted using a nitrogen bomb at 600 psi in buffer containing 25 mM Tris-HCl (pH 8.0), 25 mM KCl, 25 mM MgCl_2_, 5 mM DTT, 0.05 mM spermine, 2 mM spermidine, 1% Triton X-100, 2% polyoxyethylene 10 tridecyl ether. Lysates were cleared at 40,000×*g* prior to sucrose gradient centrifugation. Gradients were made in the above buffer (minus detergents) at 25%–45% sucrose with a 10% sucrose step on top. Sucrose gradients were overlaid with cleared cell lysate and centrifuged at 100,000 × *g* for 18 h. Fractions were collected down the gradients and monitored by SDS-PAGE and RNA gel staining. Chloroplast ribosomes and 80S cytosolic ribosomes partially copurify, so gradient fractions containing detectable cytosolic ribosomes were omitted from further processing. Fractions containing chloroplast ribosomes were then diluted in sucrose-free buffer (as above, minus detergents), and collected by ultracentrifugation at 250,000×*g*. Ribosome pellets were resuspended (buffer as above, minus detergents), snap frozen in liquid nitrogen, and stored at −80 °C until use.

### Electron microscopy.

Ribosomes, or ribosomes that had been incubated with PSRP-7 antibody, were applied to 300-mesh copper grid covered with a continuous carbon substrate that had been plasma cleaned for 20 s using a Fischione model 1020 plasma cleaner (E.A. Fischione Instruments, http://www.fischione.com/). Grids were blotted and plunged into liquid ethane through the use of a Vitrobot (http://www.vitrobot.com; FEI Company, http://www.fei.com). The Leginon system [60,61] was used for the automated acquisition of images. All images were collected on a Philips Tecnai F20 (FEI Company) operating at 120 keV equipped with a Gatan cryospecimen holder (http://www.gatan.com). Images used for reconstruction were collected under low-dose conditions (<11 e^−^/Å^2^) to a 4k × 4k Tietz CCD camera (Tietz Video and Image Processing Systems, http://www.tvips.com) at a magnification of 50,000× corresponding to a pixel size of 2.263 Å.

### Image processing and reconstruction.

ACE (Automated CTF Estimation) software was used to calculate the contrast transfer function (CTF) of micrographs [62]. Of 392 defocus pairs, 324 near-to-focus micrographs, with a defocus range from 0.7 to 2.2 μM under focus, contributed particles to the reconstruction. A small set of particles (8,882) were hand picked using the BOXER function of EMAN software [63]. These particles were used to construct a template for automated particle picking, and were also used in a reconstruction and refinement. This initial refinement used a bacterial ribosome crystal structure (1PNX and 1PNY [64]) as a starting model, and proceeded for six rounds over which the angular increment for projections was decreased from 10° to 6°. After automated particle picking on far-from-focus micrographs, a total of 101,512 particles were selected from near-to-focus pairs, and CTF was corrected by flipping the phases. The entire dataset was subjected to one round of refinement using the map calculated from the small dataset as a starting model. CORAN analysis and hierarchical clustering using SPIDER [65] then allowed for classification of the dataset, and separation of intact chloroplast ribosome images from dissociated subunit images (see Figure S1B). A total of 42,934 particles were used in the final reconstruction and refinement that produced the map presented here. EMAN refinement proceeded for 12 rounds, with a decreasing angular increment for projections from 8° to 4°. The resolution of the map presented here was estimated from the Fourier Shell Correlation curve using a 0.5 cutoff value (Figure S2).

Processing of antibody-bound ribosomes proceeded as above. Two hundred thirty-two of 416 micrographs, with a defocus range from 1.2 to 2.5 μM under focus, contributed particles the reconstruction, and an initial pool of 44,748 particles were subjected to one round of refinement in EMAN using the map from the small dataset described above as a starting model. A total of 14,866 particles passed through the CORAN analysis and hierarchical clustering, and were subjected to further refinement. Refinement was productive only through an angular increment for projections of 6°. The resolution of the map presented here was estimated from a Fourier Shell Correlation curve using a 0.5 cutoff, and is 19.4 Å (Figure S2).

### Structure analysis.

Normal mode flexible fitting (NMFF) [66,67] was used to fit E. coli ribosome crystal structure data (RCSB Protein Data Bank [PDB] IDs 1VS7 and 1VS8 [68]) to the chloroplast ribosome map. The large subunit proteins that do not have chloroplast ribosome homologs, and the small subunit proteins with large chloroplast-unique domains, were omitted from the fitting, and could be reintroduced at the all-atom model fitting stage. Individual chains were treated as phosphate or c-alpha atoms only (coarse-grained model), and each protein chain was treated as a rigid block. A maximum displacement of 2 Å per iteration was allowed, and motions were considered along ten degrees of freedom (the lowest frequency normal modes from the elastic network normal mode calculations). After over 150 iterations, NMFF brought the correlation coefficient of the fit from 0.38 (a rough hand docking was used as a starting position) to 0.56 for the coarse-grained model. The final all-atom model was constructed by a rigid-body fitting of 1VS7 and 1VS8 to the final coarse-grained model, and yielded a structure with 0.664 correlation with the chloroplast ribosome map. Further refinements allowing additional flexibility in the rRNAs were explored; however, little improvement over the initial fit was achieved. S1 position on the bacterial ribosome was recreated using differences between PDB ID 2AVY and an E. coli cryoEM reconstruction [29]. Molecular graphics images were produced using UCSF Chimera [69].

### UV cross-linking.

E. coli ribosomes for cross-linking were prepared via sucrose gradient separation similar to that described for chloroplast ribosomes, with a few modifications. Late log-phase BL21 cells were broken via freeze/thaw in buffer containing 10 mM Tris-HCl (pH 8.0), 50 mM KCl, 10 mM MgCl_2_, 2 mM DTT, 25 mM EGTA, and 1.6 μg/μl lysozyme. Gradients used for ribosome separation were 10%–40% sucrose in 25 mM Tris-HCl (pH 8.0), 25 mM KCl, 25 mM MgCl_2_. All other steps were as described above. RNA was transcribed using T7 RNA polymerase with the incorporation of [α-P32]-uridine 5′-triphosphate. The entire 91-nt *psbA* 5′ UTR in its unprocessed form was used in this experiment. UV cross-linking reactions were carried out in the presence of 150 mM Tris-HCl (pH 7.0), 250 mM KCl, 25 mM MgCl_2_, 25 mM DTT. Reactions were exposed to 2 × 600 mJ of UV radiation, after which the RNA was digested prior to separation of ribosomal proteins via denaturing SDS-PAGE. Gels were stained with Coomassie Brilliant Blue to visualize protein bands and then exposed to phosphorescent screens to visualize radiolabeled bands.

### Mass spectrometry.

Gel slices were cut from SDS-PAGE gels, and trypsinized peptides were prepared from the gel slices according to [70]. Mass spectrometry was performed as described in [71].

## Supporting Information

Figure S1 Two-Dimensional Views of the Chloroplast Ribosome (A) A representative field of view taken using cryoEM shows that a good distribution of particle orientations was obtained on continuous carbon grids. Image was taken at 50,000 × magnification; scale bar is as indicated.
(B) After an initial round of projection matching, class averages (top image of each column) indicated that particle data were heterogeneous. Reference-free classification and hierarchical clustering were used to identify self-consistent subclasses in particles matching each projection (middle three images in each column). Only subclasses containing particles corresponding to whole, assembled chloroplast ribosomes were allowed to proceed in refinement (center image in each column). Numbers indicate percentage of particles from each shown class that were clustered to each subclass.
(C) Images obtained by averaging chloroplast ribosome particles found in identical orientations (top row) are shown for comparison with two-dimensional projection images of an E. coli 70S ribosome in similar orientations (bottom row). Areas that appear to have additional density on the chloroplast ribosome compared to the bacterial ribosome are circled in black.
(1.2 MB AI).

Figure S2 Fourier Shell Correlation Curve Used for Resolution Determination A 0.5 Fourier Shell Correlation cutoff was applied to both the unliganded chloroplast ribosome map (solid line) and the PSRP-7 antibody-bound chloroplast ribosome map (dashed line). The resolutions presented for the structures are 15.5 Å for the unliganded map, and 19.4 Å for the antibody-bound map.
(239 KB AI).

Figure S3 Predicted Secondary Structure Diagram of the C. reinhardtii 16S rRNA Blue numbering indicates total position along the 16S rRNA. Helices colored in gray are lacking compared to E. coli 16S sequence and secondary structure prediction. Secondary structure diagram has been adapted from the Comparative RNA Web Site (http://www.rna.ccbb.utexas.edu).
(480 KB AI).

Figure S4 Predicted Secondary Structure Diagram of the C. reinhardtii 23S rRNA Green labels mark the individual pieces of the large subunit rRNA in C. reinhardtii chloroplast (7S, 3S, 23Sγ, and 23Sδ). Blue numbering indicates total position along the 23S rRNA. Helices colored in gray are lacking compared to E. coli 23S sequence and secondary structure prediction. Helix in checked box is extended in chloroplast compared to E. coli. Secondary structure diagram has been adapted from the Comparative RNA Web Site (http://www.rna.ccbb.utexas.edu).
(924 KB AI).

Figure S5 Head of the Chloroplast Ribosome Is Tilted Away from the Large Subunit Compared to E. coli
This shift results in a slight lift of the beak. Head and central protuberance areas of the (A) chloroplast ribosome and (B) E. coli ribosome are shown.
(1.7 MB AI).

Table S1 Protein Composition of Chloroplast and Bacterial Small Ribosomal Subunits Bold type indicates proteins with substantial size difference between bacteria and chloroplast; these proteins are discussed further in the text. Grey type indicates proteins that were not identified via proteomics as being a part of the chloroplast ribosome.
(20 KB XLS)

Table S2 Protein Composition of Chloroplast and Bacterial Large Ribosomal Subunits See Table S1 for specifics.
(22 KB XLS)

Video S1 Three-Dimensional Structure of the C. reinhardtii Chloroplast Ribosome at 15.5 Å The large subunit of the ribosome is colored lighter green, and the small subunit is darker green. Chloroplast-unique structures are found on the small subunit of the ribosome. The largest chloroplast-unique structure emerges from the head and neck region, and extends along and above the platform of the small subunit; another makes connections between the beak, head, and shoulder of the small subunit of the ribosome. See text for detailed discussion of structure, and reconstruction and refinement particulars.
(5.3 MB MOV)

### Accession Numbers

The RCSB Protein Data Bank (http://www.rcsb.org/pdb/home/home.do) accession numbers for E. coli ribosome proteins discussed in this paper are 1VS7, 1VS8, and 2AVY.

## Abbreviations

cryoEM: cryo-electron microscopy
IRES: internal ribosome entry site
mRNP: mRNA–protein complex
PSRP: plastid-specific ribosomal protein
S-D: Shine-Dalgarno
UTR: untranslated region
UV: ultraviolet
