# Supplementary material for: Structure of the Chloroplast Ribosome: Novel Domains for Translation Regulation
Source: PLoS Biol. 2007 Aug 7;5(8):e209. doi: 10.1371/journal.pbio.0050209 (PMC1939882; doi:10.1371/journal.pbio.0050209)
Supplement: Supplementary file 2 [file pbio.0050209.sg002.pdf]

Manuell Figure S2

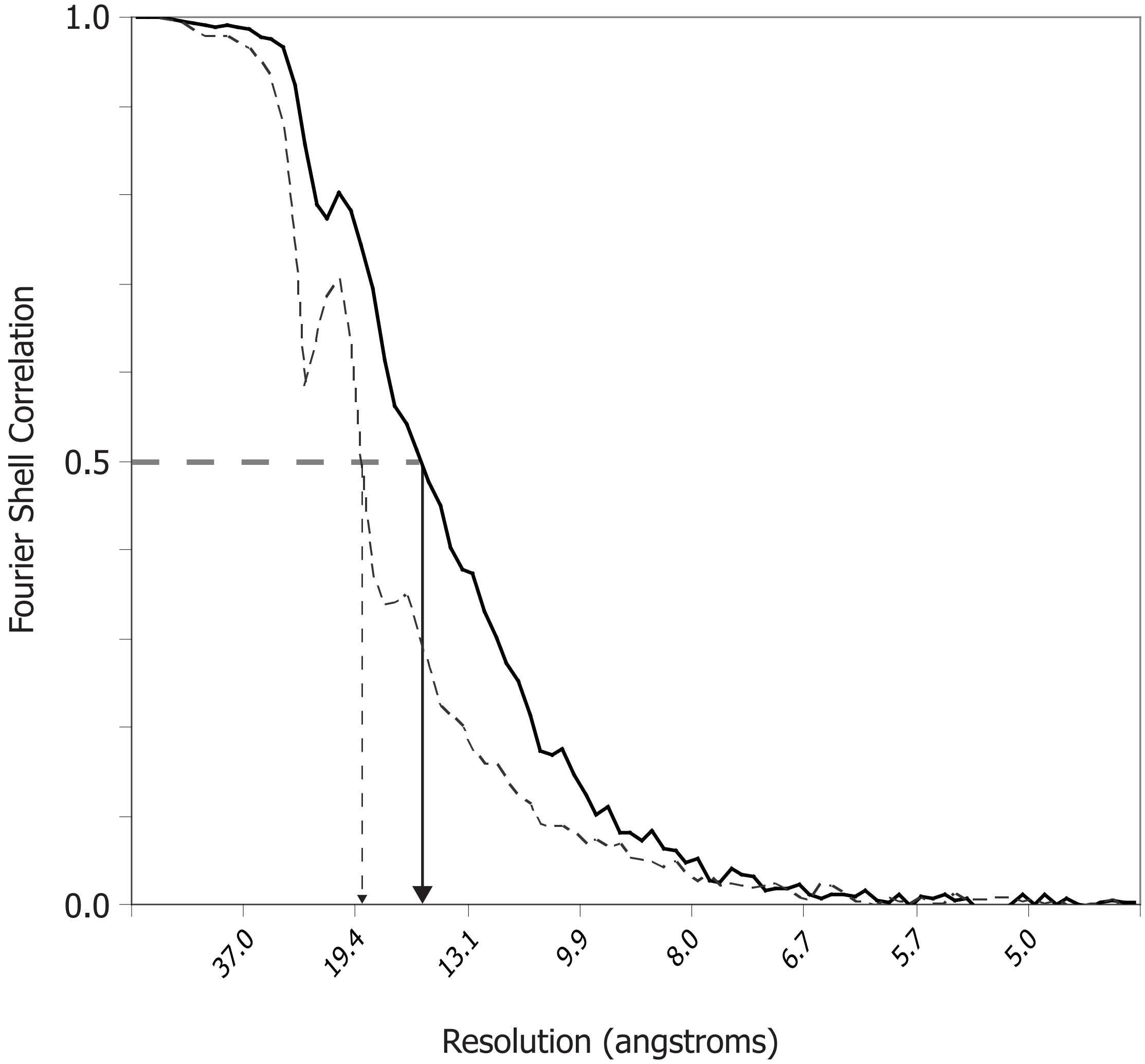

Figure S2. Fourier Shell Correlation curves used for resolution determination. A 0.5 FSC cutoff was applied to both the unliganded chloroplast ribosome map (solid line) and the PSRP-7 antibody-bound chloroplast ribosome map (dashed line). The resolutions presented for the structures are 15.5 angstroms for the unliganded map, and 19.4 angstroms for the antibody-bound map.
