# Supplementary material for: Structure of the Chloroplast Ribosome: Novel Domains for Translation Regulation
Source: PLoS Biol. 2007 Aug 7;5(8):e209. doi: 10.1371/journal.pbio.0050209 (PMC1939882; doi:10.1371/journal.pbio.0050209)

Manuell Figure S4

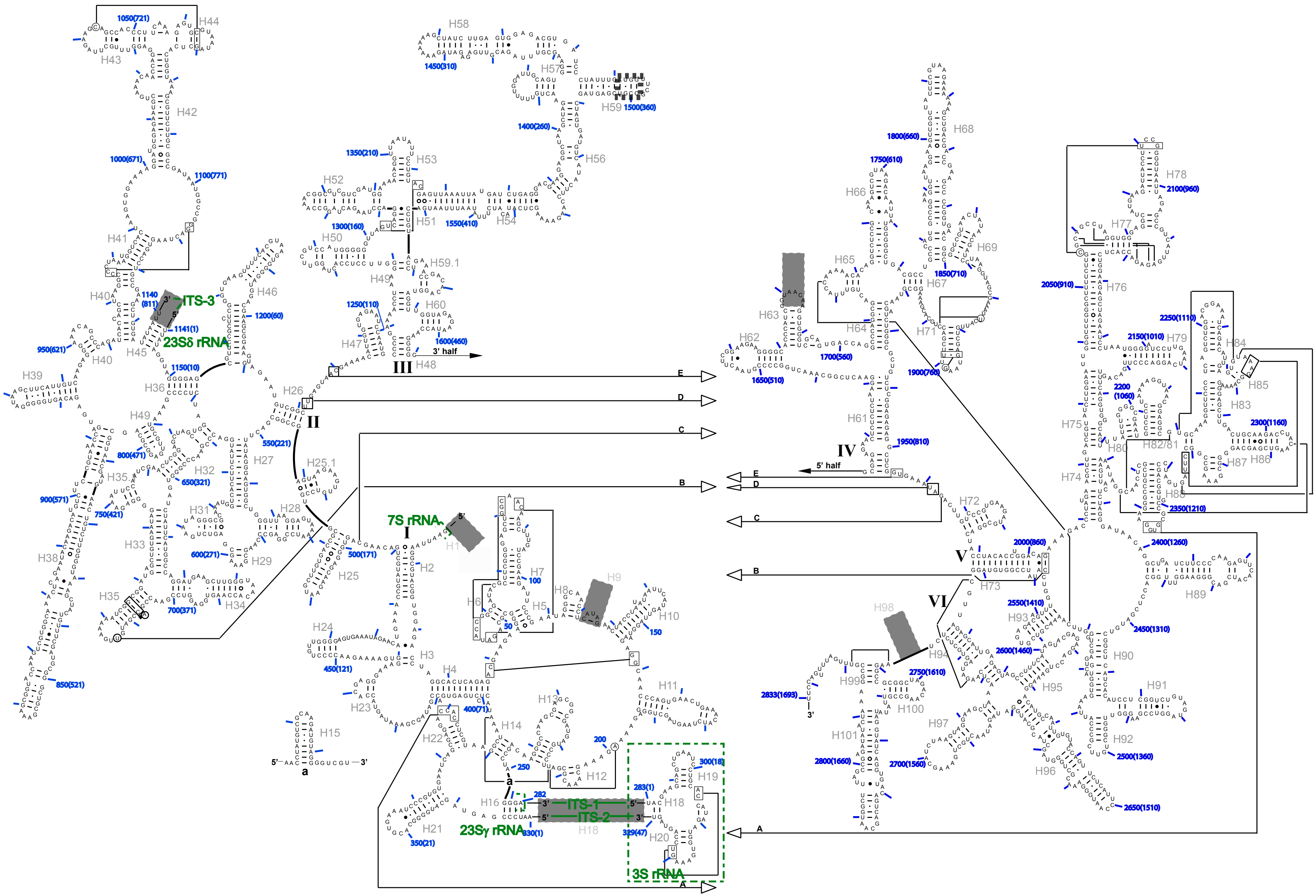

Supplement: Supplementary file 4 [file pbio.0050209.sg004.pdf]
